# A Dose–Response Study of Arsenic Exposure and Global Methylation of Peripheral Blood Mononuclear Cell DNA in Bangladeshi Adults

**DOI:** 10.1289/ehp.1206421

**Published:** 2013-09-06

**Authors:** Megan M. Niedzwiecki, Megan N. Hall, Xinhua Liu, Julie Oka, Kristin N. Harper, Vesna Slavkovich, Vesna Ilievski, Diane Levy, Alexander van Geen, Jacob L. Mey, Shafiul Alam, Abu B. Siddique, Faruque Parvez, Joseph H. Graziano, Mary V. Gamble

**Affiliations:** 1 Department of Environmental Health Sciences,; 2 Department of Epidemiology, and; 3 Department of Biostatistics, Mailman School of Public Health, Columbia University, New York, New York, USA; 4 Lamont-Doherty Earth Observatory of Columbia University, Palisades, New York, USA; 5 Columbia University Arsenic Project in Bangladesh, Dhaka, Bangladesh

## Abstract

Background: Several studies employing cell culture and animal models have suggested that arsenic (As) exposure induces global DNA hypomethylation. However, As has been associated with global DNA hypermethylation in human study populations. We hypothesized that this discrepancy may reflect a nonlinear relationship between As dose and DNA methylation.

Objective: The objective of this study was to examine the dose–response relationship between As and global methylation of peripheral blood mononuclear cell (PBMC) DNA in apparently healthy Bangladeshi adults chronically exposed to a wide range of As concentrations in drinking water.

Methods: Global PBMC DNA methylation, plasma folate, blood *S*-adenosylmethionine (SAM), and concentrations of As in drinking water, blood, and urine were measured in 320 adults. DNA methylation was measured using the [^3^H]-methyl incorporation assay, which provides disintegration-per-minute (DPM) values that are negatively associated with global DNA methylation.

Results: Water, blood, and urinary As were positively correlated with global PBMC DNA methylation (*p* < 0.05). In multivariable-adjusted models, 1-μg/L increases in water and urinary As were associated with 27.6-unit (95% CI: 6.3, 49.0) and 22.1-unit (95% CI: 0.5, 43.8) decreases in DPM per microgram DNA, respectively. Categorical models indicated that estimated mean levels of PBMC DNA methylation were highest in participants with the highest As exposures.

Conclusions: These results suggest that As is positively associated with global methylation of PBMC DNA over a wide range of drinking water As concentrations. Further research is necessary to elucidate underlying mechanisms and physiologic implications.

Citation: Niedzwiecki MM, Hall MN, Liu X, Oka J, Harper KN, Slavkovich V, Ilievski V, Levy D, van Geen A, Mey JL, Alam S, Siddique AB, Parvez F, Graziano JH, Gamble MV. 2013. A dose–response study of arsenic exposure and global methylation of peripheral blood mononuclear cell DNA in Bangladeshi adults. Environ Health Perspect 121:1306–1312; http://dx.doi.org/10.1289/ehp.1206421

## Introduction

An estimated 70 million people in Bangladesh are chronically exposed to arsenic (As)-contaminated drinking water at levels above the World Health Organization (WHO) limit of 10 μg/L (Loewenberg 2007). Exposure to As has been associated with cancers of the skin, lung, bladder, liver, and kidney (Navarro Silvera and Rohan 2007), hypertension and cardiovascular disease (Chen CJ et al. 1995; Chen Y et al. 2011; Tseng et al. 2003), respiratory outcomes (Chattopadhyay et al. 2010; Dauphiné et al. 2011; Parvez et al. 2010), and reduced cognitive function in children (Hamadani et al. 2011; Wasserman et al. 2004). The mechanism(s) responsible for the pleiotropic health effects of As are not completely understood.

DNA methylation involves the covalent addition of a methyl group from *S*-adenosylmethionine (SAM) to the 5´ position of cytosine bases in CpG dinucleotides (Zhao et al. 1997). SAM biosynthesis is regulated by one-carbon metabolism, a biochemical pathway for the methylation of numerous substrates that is dependent on folate for the recruitment of methyl groups from serine (Chiang et al. 1996). Methylation of CpG dinucleotides is associated with gene silencing: CpGs in intergenic regions and repetitive elements are usually methylated (Robertson 2001), whereas CpGs in gene promoter regions of transcriptionally active genes are generally less methylated than those in other regions (Razin and Kantor 2005). Global DNA hypomethylation is associated with genomic instability, including loss of heterozygosity (Matsuzaki et al. 2005), aneuploidy (Dodge et al. 2005), and chromosomal alterations (Schulz et al. 2002), and is commonly observed in tumors and transformed cells (Gaudet et al. 2003). In addition, the ENCODE Project Consortium (2004) has highlighted important regulatory roles of methylation of transposable elements and other noncoding regions of the genome (previously coined “junk DNA”) in cell-type–dependent transcriptional regulation (Bernstein et al. 2012; Thurman et al. 2012).

Evidence based on *in vitro* and *in vivo* models suggests that chronic As exposure induces global DNA hypomethylation, in conjunction with hypermethylation of promoter regions of tumor suppressor genes (Benbrahim-Tallaa et al. 2005; Chen et al. 2004; Reichard et al. 2007; Ren et al. 2011). On the basis of this literature, we initially hypothesized that, in a population chronically exposed to As-contaminated drinking water, As exposure would be associated with a reduction in global methylation of DNA, thus providing a mechanistic link between As exposure and increased cancer risk. We further hypothesized that this would be exacerbated by folate deficiency. However, in our first population-based study on this subject, contrary to our *a priori* hypotheses, chronic As exposure was positively associated with global methylation of peripheral blood leukocyte DNA among subjects with sufficient folate levels (Pilsner et al. 2007). Although these findings were unanticipated, the positive association between As exposure and global DNA methylation in humans has been replicated by our group (Pilsner et al. 2009, 2012) and by others (Kile et al. 2012; Lambrou et al. 2012; Majumdar et al. 2010). We hypothesized that the discrepancy between experimental and human studies is related to the dose and duration of As exposure: High acute As exposures might result in DNA hypomethylation, whereas a nonlinear pattern of association might be observed within the range of As exposures in human populations, many of whom have been exposed to As for decades.

The primary objective of this analysis was to evaluate the relationship between As exposure and global methylation of peripheral blood mononuclear cell (PBMC) DNA. We used data from the Folate and Oxidative Stress (FOX) study (Hall et al. 2013), which selected study participants who had a wide range of As exposures; the primary objective of that study was to assess the relationship between As exposure and oxidative stress. The FOX study provided a unique opportunity to examine the dose–response relationship between As exposure and global DNA methylation in a population with higher levels of As exposure than previous study populations.

## Subjects and Methods

*Eligibility criteria and study design*. For the FOX study, 379 men and women between 35 and 65 years of age were recruited between April 2007 and April 2008 in Araihazar, Bangladesh. Participants were selected on the basis of their exposure to well water As (wAs), so that the final study sample represented the full range of wAs concentrations in the region. We aimed to recruit 75 participants from each of five exposure categories (group A, 0–10 μg/L; group B, 10–100 μg/L; group C, 100–200 μg/L; group D, 200–300 μg/L; and group E, > 300 μg/L) but had difficulty recruiting participants in the highest exposure categories because households using wells that tested positive for As (i.e., > 50 μg/L, the Bangladesh standard) were encouraged to switch to lower-As wells (Chen et al. 2007). Thus, the final number of participants for the five categories were as follows: group A, *n* = 76; group B, *n* = 104; group C, *n* = 86; group D, *n* = 67, and group E, *n* = 45. A random sample in this region would have likely yielded 40–50% of participants who consumed wAs ≤ 50 μg/L. Participants must have been drinking from the same well for a minimum of 3 months prior to recruitment. Participants were excluded if they were pregnant; were currently taking nutritional supplements (or had done so within the past 3 months); or had known diabetes or cardiovascular or renal disease.

Oral informed consent was obtained by our Bangladeshi field staff physicians, who read an approved consent form to the study participants. This study was approved by the institutional review boards of the Bangladesh Medical Research Council and of Columbia University Medical Center.

*Analytic techniques*. Sample collection and handling. Blood samples were drawn and processed immediately at our field clinic laboratory in Araihazar. Blood samples were centrifuged at 3,000 × *g* for 10 min at 4°C, and buffy coat and plasma were separated from red cells. Aliquots of blood and plasma were stored at –80°C. Urine samples were collected in 50-mL acid-washed polypropylene tubes and frozen at –20°C. Blood, plasma, and urine samples were transported to Dhaka, Bangladesh, on dry ice and stored at –80°C. Samples were then shipped, frozen on dry ice, to Columbia University for analysis.

Water As. Field sample collection and laboratory procedures have been described in detail (Cheng et al. 2004; van Geen et al. 2005). Briefly, at the recruitment visit of the FOX study, new water samples were collected in 20-mL polyethylene scintillation vials and acidified to 1% with high-purity Optima hydrochloric acid (Fisher Scientific, Pittsburg, PA, USA) at least 48 hr before analysis (van Geen et al. 2007). Water samples, diluted 1:10 and spiked with germanium to correct fluctuations in instrument sensitivity, were analyzed by high-resolution inductively coupled plasma mass spectrometry (ICP-MS). A standard (As concentration of 51 μg/L) was run multiple times in each batch, with intraassay and interassay coefficients of variation (CVs) for this standard of 6.0% and 3.8%, respectively.

Total urinary As and urinary creatinine. Urinary As (uAs) metabolites were speciated using HPLC separation of arsenobetaine (AsB), arsenocholine (AsC), arsenate (As^V^), arsenite (As^III^), monomethylarsonic acid (MMA^III^ + MMA^V^), and dimethylarsinic acid (DMA^V^), followed by detection using ICP-MS (Vela et al. 2001). Total uAs was calculated by summing the concentrations of As^V^, As^III^, MMA, and DMA; AsC and AsB were not included in total As. The limit of detection (LOD) for each uAs metabolite was 0.1 μg/L. Arsenic levels in urine were determined with and without adjustment for urinary creatinine, which was analyzed by a colorimetric assay based on the Jaffe reaction (Slot 1965). The intraassay CVs were 4.5% for As^V^, 3.8% for As^III^, 1.5% for MMA, and 0.6% for DMA; the interassay CVs were 10.6% for As^V^, 9.6% for As^III^, 3.5% for MMA, and 2.8% for DMA.

Total blood As. Total blood As (bAs) was analyzed using a PerkinElmer Elan DRC II ICP-MS equipped with an AS 93+ autosampler (PerkinElmer, Shelton, CT, USA), with an LOD of 0.1 μg/L, as previously described (Hall et al. 2006). The intraassay and interassay CVs were 3.2% and 5.7%, respectively.

PBMC DNA isolation. PBMCs were isolated from fresh blood samples in the field clinic laboratory in Araihazar. To isolate PBMCs, 4 mL Ficoll solution was added to a tube containing 4 mL blood (with serum removed) and 11 mL phosphate-buffered saline (PBS). Tubes were centrifuged at 400 × *g* for 30 min, and the mononuclear cell layer was extracted and washed with PBS. Cells were sedimented by centrifuging at 200 × *g* for 10 min, and the cell pellet was resuspended with 4 mL lysis solution and 50 μL proteinase K solution. PBMC lysates were stored at 4°C until shipment to Columbia University. PBMC DNA was isolated from 4 mL PBMC lysate using 1 mL Protein Precipitation Solution (5-Prime, New York, NY, USA) and standard isopropanol extraction following the manufacturer’s protocol. DNA was stored at –20°C until further analysis.

Global DNA methylation. Global DNA methylation was measured using the [^3^H]-methyl incorporation assay (Balaghi and Wagner 1993) as previously described (Pilsner et al. 2007). The assay employs ^3^H-labeled SAM and SssI methylase to add ^3^H-labeled methyl groups to unmethylated CpG sequences. Thus, disintegration-per-minute (DPM) values are negatively associated with global DNA methylation. Samples were run in duplicate, and each run included a blank (mixture including all reaction components except SssI enzyme), hypomethylated control (HeLa cell DNA), and positive control (DNA extracted from whole blood sample). PicoGreen dsDNA Quantitation Reagent (Molecular Probes, Eugene, OR, USA) was used to quantify the exact amount of double-stranded DNA (dsDNA) used in each reaction. The mean DPM values from the duplicate samples were expressed per microgram DNA, as determined by PicoGreen. The intraassay and interassay CVs were 3.4% and 10.4%, respectively.

Plasma folate. Plasma folate was analyzed by radioprotein binding assay (SimulTRAC-S; MP Biomedicals, Orangeburg, NY, USA). This method requires heating plasma to 100°C to denature endogenous binding substances. To determine folate concentration, we used folic acid as pteroylglutamic acid for calibration, and its ^125^I-labeled analog was used as the tracer. The intraassay and interassay CVs were 6% and 14%, respectively.

Plasma homocysteine. Total plasma homocysteine (Hcys) was measured using high-performance liquid chromatography (HPLC) with fluorescence detection, as previously described (Pfeiffer et al. 1999). The intraassay and interassay CVs were 2% and 9%, respectively.

Blood SAM. SAM was measured in whole blood as described previously (Poirier et al. 2001). SAM was detected at 254 nm using a 996 Photodiode Array ultraviolet absorbance detector (Waters Inc., Milford, MA, USA) and quantified relative to standard curves (Sigma Chemical Co., St. Louis, MO, USA). The interassay CV was 9.6%.

*Statistical methods*. Descriptive statistics (means ± SDs) were calculated for the overall sample. We used Spearman correlations (for continuous variables) and the Wilcoxon rank-sum test (for dichotomous variables) to examine bivariate associations between As variables and other covariates with [^3^H]-methyl incorporation. Certain confounders (sex, age, and ever versus never smoking cigarettes) were selected based on biologic plausibility and our previous studies (Pilsner et al. 2007). Other potential confounders [body mass index (BMI), years of education, plasma folate, plasma vitamin B_12_, ever using betel nuts, and television ownership] were selected based on bivariate associations with markers of As exposure and [^3^H]-methyl incorporation in this data set, but none met our criteria for inclusion in the final models (*p* > 0.20 for associations of the potential confounder with both the exposure and the outcome).

Urinary As was adjusted for urinary creatinine using the residual method. To estimate these adjusted values, we constructed linear regression models with log-transformed urinary creatinine as the predictor of log-transformed uAs. The residuals from this model were added back to the mean log-transformed uAs and exponentiated to get the final urinary creatinine–adjusted uAs values. The urinary creatinine–adjusted uAs variable was used for all analyses involving uAs.

We used locally estimated scatter plot smoothing (LOESS) curves of unadjusted associations between As exposure variables and [^3^H]-methyl incorporation to visually identify potential nonlinear or nonmonotonic relationships. We used a SAS macro (SAS Institute Inc., Cary, NC, USA) for restricted cubic splines to estimate associations between markers of As exposure and [^3^H]-methyl incorporation adjusted for sex, age, and smoking status (Desquilbet and Mariotti 2010). A restricted cubic spline function represents the sum of piecewise cubic polynomial splines with continuity and constraints at 3–5 specified knots on the continuous exposure variables (Desquilbet and Mariotti 2010). A Wald chi-square test with *K* – 2 degrees of freedom, where *K* is the number of knots and *K* – 2 is the number of spline variables, was used to test the null hypothesis of linear relationship between the exposure and outcome variables. Failure to reject the null hypothesis of linearity would support the use of linear regression to estimate associations between markers of As exposure (modeled as untransformed continuous variables) and [^3^H]-methyl incorporation.

We also estimated associations by modeling As exposure using categorical variables. Water As categories were created with the reference category reflecting the Bangladeshi drinking water standard for As of 50 μg/L: 0–50 μg/L, 50–100 μg/L, 100–200 μg/L, 200–300 μg/L, and 300–700 μg/L. Urinary As was first adjusted for urinary creatinine using the residual method; quintiles of uAs exposures were then constructed based on these adjusted values. Blood As was categorized based on quintiles. Separate general linear models were constructed with each categorized As exposure variable as the predictor and [^3^H]-methyl incorporation as the outcome, with sex, age, and ever smoking included in the model as covariates. Least-squares means for [^3^H]-methyl incorporation were calculated for each level of each As exposure variable, assuming the mean value for age (43.175 years) and a value of 0.5 for the dichotomous covariates sex and ever smoking.

[^3^H]-Methyl incorporation values were excluded from the analysis if duplicate assays had CVs > 15% (*n* = 48), if < 10 μg/mL of DNA was used in the assay (*n* = 8), or if DPM values were extreme outliers, defined as values that exceeded the 75th percentile of the DPM values by more than three interquartile ranges (*n* = 2). In addition, we were unable to extract DNA from 1 lysate, leaving a final data set of 320 lysates for the analyses. All statistical analyses were conducted using SAS (version 9.2; SAS Institute Inc.); statistical tests were two sided with a significance level of 0.05.

## Results

The characteristics of the study population are shown in Table 1. The mean age was 43.2 years, and there were roughly equal numbers of males and females. Based on cutoffs of < 9.0 nmol/L for folate deficiency and > 13 μmol/L for hyperhomocysteinemia, 31.3% and 17.5% of the participants were classified as having folate deficiency and hyperhomocysteinemia, respectively. More stringent cutoffs (10.4 μmol/L for females and 11.4 μmol/L for males) would result in classification of 29% of the participants as having hyperhomocysteinemia. Water As concentrations ranged from 0.4 to 700 μg/L, with a mean of 145 μg/L. By design, wells that exceeded the Bangladeshi standard of 50 μg/L were the primary drinking water source for 70.9% of participants.

**Table 1:** Demographic and analytic measures for study participants (*n* = 320).

Baseline variable	Mean ± SD (range) or *n* (%)
Demographic
Age (years)	43.2 ± 8.3 (30–63)
Male	159 (49.7)
BMI (kg/m^2^)	20.3 ± 3.5 (13.8–35.3)
Underweight (BMI < 18.5 kg/m^2^)	112 (35.1)
Ever smoked cigarettes	123 (38.4)
Ever used betel nuts	142 (44.4)
Owned television	185 (57.8)
Analytic measure
wAs (μg/L)^*a*^	145 ± 123 (0.4–700)
wAs > 50 μg/L^*a*^	226 (70.9)
uAs (μg/L)	213 ± 242 (2–1,800)
Urinary creatinine (mg/dL)	54.5 ± 43.7 (4.3–223.5)
uAs [creatinine adjusted (μg/L)]	167 ± 122 (10–548)
bAs (μg/L)	13.9 ± 9.8 (1.2–57.0)
Plasma folate (nmol/L)^*a*^	12.6 ± 7.0 (2.4–60.6)
Folate deficient (folate < 9 nmol/L)^*a*^	100 (31.3)
Plasma homocysteine (μmol/L)	11.2 ± 12.0 (3.0–165.4)
Hyperhomocysteinemia (homocysteine > 13 μmol/L)	56 (17.5)
Blood SAM (μM)^*b*^	1.29 ± 0.50 (0.44–3.38)
[^3^H]-Methyl incorporation (DPM/μg DNA)	149,123 ± 23,932 (61,398–215,666)
^***a***^*n *= 319. ^***b***^*n *= 312.	

All As variables (wAs, bAs, uAs, and creatinine-adjusted uAs) were negatively correlated with [^3^H]-methyl incorporation (Table 2), indicating that As exposure was positively correlated with PBMC DNA methylation. Spearman correlation coefficients were similar between groups when stratified by folate deficiency (plasma folate < 9.0 nmol/L) (data not shown), suggesting that folate status did not modify associations between As exposure markers and [^3^H]-methyl incorporation in this sample. Correlations also were similar between groups defined using more stringent definitions of folate deficiency (plasma levels < 8.0 nmol/L, *n* = 69; < 7.0 nmol/L, *n* = 38) (data not shown). We also examined correlations of As and [^3^H]-methyl incorporation with blood SAM, which has been hypothesized to mediate the relationship between As exposure and DNA methylation (Mass and Wang 1997). Although blood SAM was negatively correlated with [^3^H]-methyl incorporation, As was not correlated with blood SAM (Table 2). In addition, methylated metabolites of As were not correlated with [^3^H]-methyl incorporation (data not shown). We did not observe significant correlations between plasma folate and [^3^H]-methyl incorporation or blood SAM, or between age and [^3^H]-methyl incorporation (Table 2).

**Table 2:** Spearman correlation coefficients for arsenic variables, [^3^H]‑methyl incorporation in PBMC DNA (DPM/μg DNA), blood SAM, and plasma folate (*n* = 320).

	bAs	uAs	uAs (Cr adj)	DPM/μg DNA	Blood SAM^*a*^	Plasma folate^*b*^	Age
wAs^*a*^	0.75^#^	0.63^#^	0.75^#^	–0.14*	–0.03	–0.04	–0.01
bAs		0.67^#^	0.93^#^	–0.13*	–0.03	–0.06	0.00
uAs			0.71^#^	–0.08	0.00	0.03	–0.02
uAs (Cr adj)				–0.12*	–0.04	0.00	0.01
DPM/μg DNA					–0.12*	0.03	0.07
Blood SAM^*a*^						–0.07	0.13*
Plasma folate^*b*^							–0.12*
Cr adj, creatinine adjusted.^***a***^*n *= 312. ^***b***^*n *= 319. *p < 0.05. ^#^*p *< 0.0001.							

The shapes of the LOESS curves suggested that [^3^H]-methyl incorporation decreased linearly with increasing levels of wAs, bAs, and uAs (data not shown). Using restricted cubic spline functions with 5 knots, we observed a similar decrease in [^3^H]-methyl incorporation as As levels increased (data not shown). Tests of departures from the null hypothesis of linear dose–response relations were not statistically significant for any of the As predictors (all *p*-values > 0.30).

Unadjusted linear regression models of wAs, uAs, or bAs as predictors of [^3^H]-methyl incorporation indicated negative associations between As exposure and [^3^H]-methyl incorporation that were statistically significant for wAs [β = –28.2; 95% confidence interval (CI): –49.5, –6.8, *p* = 0.01] and borderline significant for uAs (β = –20.2; 95% CI: –41.9, 1.4, *p* = 0.07) and bAs (β = –254.5; 95% CI: –523.5, 14.6, *p* = 0.06) (Table 3). Estimates for the difference in mean DPM estimates associated with a 1-unit increase in As exposure adjusted for sex, age, ever smoking, and urinary creatinine (for uAs), were similar to unadjusted estimates for wAs (β = –27.6; 95% CI: –49.0, –6.3; *p* = 0.01) and uAs (β = –22.1; 95% CI: –43.8, –0.5; *p* = 0.045), but the association for bAs was attenuated (β = –211.7; 95% CI: –483.9, 60.5; *p* = 0.13, compared with β = –254.5; 95% CI: –523.5, 14.6, before adjustment).

**Table 3:** Estimated regression coefficients from separate linear regression models of associations between As exposure variables and [^3^H]‑methyl incorporation in PBMC DNA (*n* = 320).

Predictor	Unadjusted		Adjusted^*a*^	
	β (95% CI)	*p*-Value	β (95% CI)	*p*-Value
wAs^*b*^	–28.2 (–49.5, –6.8)	0.01	–27.6 (–49.0, –6.3)	0.01
uAs (creatinine adjusted)	–20.2 (–41.9, 1.4)	0.07	–22.1 (–43.8, –0.5)	0.05
bAs	–254.5 (–523.5, 14.6)	0.06	–211.7 (–483.9, 60.5)	0.13
^***a***^Adjusted for sex, age, and ever cigarette smoking. ^***b***^*n *= 319.				

**Figure 1:**
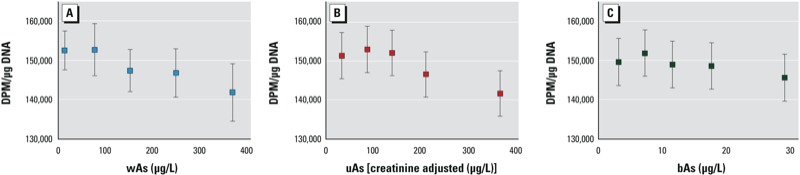
Least-squares mean values of [^3^H]‑methyl incorporation by category means of wAs (*A*), uAs (*B*), and bAs (*C*). Data points represent estimated mean DPM values and 95% CIs according to the mean As value for each As-exposure category, assuming the mean value for age (43.2 years) and a value of 0.5 for the dichotomous covariates sex and ever smoking. Categories for bAs and uAs are quintiles, whereas categories for wAs reflect study sampling categories and the Bangladeshi As standard of 50 μg/L.

We constructed linear models to estimate least-squares mean [^3^H]-methyl incorporation levels by As category, with sex, age, and ever smoking included as covariates (Figure 1). [Categories for wAs reflect study sampling categories and the Bangladeshi As standard of 50 μg/L, whereas categories for bAs and uAs are quintiles.] Mean [^3^H]-methyl incorporation levels were similar between the lowest two wAs categories (Figure 1A) and among the lowest three uAs quintiles (Figure 1B). Higher wAs- and uAs-exposure categories were associated with lower estimated least-squares mean values for [^3^H]-methyl incorporation: The estimated mean [^3^H]-methyl incorporation was 7.0% lower (95% CI: 2.2, 11.8) in the highest wAs category (300–700 μg/L) than in the 0–50 μg/L wAs referent group (*p* = 0.02; Figure 1A) and was 6.4% lower (95% CI: 2.5, 10.3) in the highest uAs quintile compared with the lowest uAs quintile (*p* = 0.02; Figure 1B). Although the pattern of association for [^3^H]-methyl incorporation by bAs quintile was similar to the pattern for wAs and uAs (Figure 1C), the least-squares means were not significantly different from one another.

## Discussion

The primary objective of this study was to assess the dose–response relationship between As exposure and global methylation of PBMC DNA in adults chronically exposed to a wide range of As in drinking water in Bangladesh. In agreement with previous findings (Pilsner et al. 2007, 2009), As exposure was positively associated with global DNA methylation of PBMC DNA. Furthermore, within the range of As exposures in our study, we did not detect a statistically significant departure from linearity in the association between As and DNA methylation. Adjusted mean DNA methylation levels were similar among the lower As-exposure categories but increased in the higher As-exposure categories, with the highest estimated mean DNA methylation levels in the highest As-exposure categories (wAs > 300 μg/L). However, we cannot rule out the possibility of nonlinearity at extremely high doses often used in animal studies [e.g., As exposures ranging from 1 to 85 ppm (1,000–85,000 μg/L)] (Reichard and Puga 2010).

In the present study, we did not find a significant correlation between age and [^3^H]-methyl incorporation. It is possible that this is attributable to the demographics of the study population. In our first study of As exposure and global DNA methylation (Pilsner et al. 2007), global DNA methylation decreased with age across the 18–29, 30–36, and 37–45 year age categories. However, global DNA methylation levels did not differ significantly between the 37–45 and 46–66 year age groups (Pilsner et al. 2007). The narrower age range of the FOX study population (30–65 years) may therefore have contributed to the lack of a significant association between global DNA methylation and age in the present study.

We did not find a correlation between plasma folate and [^3^H]-methyl incorporation. The mean plasma folate level was higher in the present study than in our first study of As exposure and global DNA methylation (Pilsner et al. 2007) (12.6 vs. 8.6 nmol/L, respectively), and a smaller proportion was folate deficient (31.3% vs. 64.6%). Folate might influence DNA methylation only under conditions in which folate is limiting. In support of this hypothesis, folic acid supplementation was not associated with higher global DNA methylation in study populations whose participants were folate sufficient at baseline (Basten et al. 2006; Jung et al. 2011). In the present study population, associations between As and DNA methylation were similar between those with and without folate deficiency, even when more stringent definitions of folate deficiency were used. However, our ability to evaluate an interaction may have been limited by the small numbers of participants with severe folate deficiency.

Other epidemiologic studies in adults have reported that associations between As exposure and global DNA methylation were modified by folate status. For example, in a study of elderly men in Massachusetts, toenail As concentrations were positively associated with Alu methylation only among men with plasma folate levels below the study median (Lambrou et al. 2012). Although this seemingly contradicts our previous finding of an association between As exposure and DNA methylation that was limited to participants with sufficient plasma folate levels (Pilsner et al. 2007), Lambrou et al. (2012) noted that plasma folate levels in our folate-sufficient group (> 9.0 nmol/L) overlapped with levels in their low-folate group (< 32 nmol/L). In the present study, all but six participants had plasma folate levels < 32 nmol/L.

Two studies have reported positive associations between prenatal As exposure and global DNA methylation. In a study of 113 mother/newborn pairs in Bangladesh, Kile et al. (2012) reported that As exposure was associated with higher methylation of LINE-1 (long interspersed nucleotide elements) in both umbilical cord blood and maternal leukocytes. In a similar study of 101 mother/newborn pairs in Bangladesh, Pilsner et al. (2012) observed that maternal As exposure was associated with increased global DNA methylation based on [^3^H]-methyl incorporation in DNA extracted from umbilical cord blood leukocytes. However, the direction of the associations between maternal As exposure and other markers of global DNA methylation [Alu (short interspersed elements), LINE-1, and LUMA (luminometric methylation assay)] differed by sex: Associations were positive in male newborns, but negative in female newborns (Pilsner et al. 2012).

Several mechanisms of As-induced epigenetic dysregulation have been proposed. For example, because SAM is required for As metabolism, Mass and Wang (1997) hypothesized that As methylation may influence DNA methylation through competition for methyl groups. Using an *in vitro* model, Reichard et al. (2007) observed that low-dose As exposure decreased SAM concentrations in keratinocytes cultured in folic acid–deficient medium. Although blood SAM was positively correlated with DNA methylation in the present study, it was not correlated with markers of As exposure. In addition, in contrast with what might be expected if there were competition between DNA methyltransferases and arsenic methyltransferase for SAM, As metabolites were not correlated with DNA methylation in our study population. Quantitatively, methylation of As and DNA consumes only a very small proportion of total SAM (Gamble and Hall 2012).

There is growing evidence that As exposure may alter posttranslational modifications of lysine residues in histone tails. For example, exposure to NaAsO_2_ (sodium meta-arsenite) led to increased global histone acetylation and more open chromatin formation in human HepG2 hepatocarcinoma cells (Ramirez et al. 2008), and exposure to trivalent inorganic arsenic (InAs^III^) led to increased H3K9 dimethylation (H3K9me2) and decreased H3K27 trimethylation (H3K27me3) in human lung carcinoma A549 cells (Zhou et al. 2008). Occupational exposure to inhalable As was significantly associated with H3K4me2 and H3K9 acetylation (H3K9ac) in histones purified from white blood cells in a study of steel workers in Italy (Cantone et al. 2011). In ongoing studies, we are evaluating associations of As exposure on global histone modifications in As-exposed Bangladeshis (Chervona et al. 2012) and whether these histone marks are also associated with global DNA methylation.

The physiologic implications of increased global DNA methylation in apparently healthy adults are unclear, given that global DNA hypomethylation has been associated with multiple diseases, including As-induced skin lesions. An increase in global DNA methylation might be associated with an increased spontaneous mutation rate: Methylated cytosines are more prone to deamination than unmethylated cytosines, which increases the likelihood of C→T transitions (Ehrlich et al. 1986). In addition, DNA methylation is associated with heterochromatic regions (Beisel and Paro 2011), and somatic mutation density in human cancer genomes have been found to be highest in heterochromatin-like domains (Schuster-Bockler and Lehner 2012).

Previous findings suggest that As-induced global DNA hypermethylation might be associated with concomitant increases in As-induced gene-specific promoter methylation of tumor suppressors or other disease-related genes. Kile et al. (2012) reported that uAs was associated with both increased LINE-1 methylation and increased *P16* promoter methylation of leukocyte DNA from umbilical cord blood and maternal blood. Promoter methylation of *P16* and *P53* tumor suppressors was elevated in both As-exposed individuals and As-associated skin cancer cases in West Bengal, India (Chanda et al. 2006), and methylation of the tumor suppressors *RASSF1A* and *PRSS3* was associated with toenail As concentrations and invasive tumor stage in bladder cancer cases in New Hampshire (Marsit et al. 2006). In women from the Argentinean Andes, uAs concentrations were positively associated with promoter methylation of *P16* and the DNA repair gene *mLH1* (Hossain et al. 2012).

We cannot dismiss the possibility that our findings may be explained by As-induced shifts in blood-cell type distributions. In our previous studies (Pilsner et al. 2007, 2009), DNA was isolated from peripheral blood leukocytes, whereas in the present study we used PBMCs. Because PBMCs are a subset of the cells present in peripheral blood leukocytes, this would reduce cell type variability to some extent. Soto-Peña et al. (2006) reported that arsenic exposure was not significantly associated with proportions of total T cells, cytotoxic T cells, B cells, or natural killer cells in PBMCs isolated from As-exposed children in Mexico. Furthermore, Wu et al. (2011) observed that average [^3^H]-methyl incorporation levels were not significantly different among DNA samples isolated from granulocytes, mononuclear cells, and white blood cells.

In the present study population, the *p*-value for the association of [^3^H]-methyl incorporation with blood As (0.13) was larger than *p*-values for corresponding associations with wAs or uAs (0.01 and 0.045, respectively). Blood As is the most proximal marker of PBMC exposure, so one might expect that bAs would have the strongest and/or most significant association with PBMC DNA methylation. Although blood As was strongly correlated with wAs (Spearman *r* = 0.75) and creatinine-adjusted uAs (Spearman *r* = 0.93), the range of concentrations of As in blood (1.2–57.0 μg/L) was much smaller than that in urine (10–548 μg/L) or water (0.4–700 μg/L), resulting in reduced statistical power to predict [^3^H]-methyl incorporation.

The present study has several limitations. First, PBMCs are not known to be direct targets of As-induced carcinogenesis. However, As trioxide is a highly effective therapeutic drug for the treatment of acute promyelocytic leukemia (Powell et al. 2010), demonstrating that As distributes to bone marrow progenitor cells and influences their cellular function (Petrie et al. 2009; Soignet et al. 1998). We do not know the extent to which methylation of PBMC DNA reflects the methylation of other target tissues. Finally, it is possible that our results can be explained by unmeasured confounding. Notably, we do not have measures of all possible contaminants in well water that may co-occur with As and potentially confound our observed associations. However, mass spectrometry data for a panel of 33 elements from well water samples from our study area indicated that only As and manganese were elevated (Cheng et al. 2004), and manganese was not associated with DNA methylation (data not shown).

## Conclusion

Arsenic exposure was positively associated with global methylation of PBMC DNA in Bangladeshi adults with a wide range of As exposures, consistent with previous studies. Furthermore, we did not observe statistical evidence of nonlinear relationships between As and global DNA methylation within the range of As exposures in our population. Future studies, in addition to investigating the influence of As on histone modifications, should evaluate the potential physiological implications of increased global DNA methylation.
